# Quaternary structures of recombinant, cellular, and serum forms of Thymidine Kinase 1 from dogs and humans

**DOI:** 10.1186/1471-2091-13-12

**Published:** 2012-06-28

**Authors:** Sharif Hanan, Kiran Kumar Jagarlamudi, Wang Liya, He Ellen, Eriksson Staffan

**Affiliations:** 1 Department of Anatomy, Physiology and Biochemistry, Swedish University of Agricultural Sciences, BMC, 575, Uppsala, S-751 23, Sweden; 2 Sino-Swed Molecular Bio-Medicine Research Institute, High-Tech Industrial Park, Shenzhen, 518057, China

**Keywords:** Recombinant Thymidine Kinase 1, Canine, Human, AZT, Serum Thymidine Kinase 1

## Abstract

**Background:**

Thymidine kinase 1 (TK1) is a salvage enzyme involved in DNA precursor synthesis, and its expression is proliferation dependent. A serum form of TK1 has been used as a biomarker in human medicine for many years and more recently to monitor canine lymphoma. Canine TK1 has not been cloned and studied. Therefore, dog and human TK1 cDNA were cloned and expressed, and the recombinant enzymes characterized. The serum and cellular forms of canine and human TK1 were studied by size-exclusion chromatography and the level of TK1 protein was determined using polyclonal and monoclonal anti-TK1 antibodies.

**Results:**

Canine TK1 phosphorylated the thymidine (dThd) analog 3'-azido-thymidine (AZT) as efficiently as it did dThd, whereas AZT phosphorylation by human TK1 was less efficient than that of dThd. Dog TK1 was also more thermostable and pH tolerant than the human enzyme. Oligomeric forms were observed with both enzymes in addition to the tetrameric and dimeric forms. Cellular TK1 was predominantly seen in dimeric and tetrameric forms, in the case of both dog TK1 from MDCK cells and human TK1 from CEM cells. Active serum TK1 was found mainly in a high molecular weight form, and treatment with a reducing agent shifted the high molecular weight complex to lower molecular weight forms with reduced total activity. Western blot analysis demonstrated a polypeptide of 26 kDa (dog) and 25 kDa (human) for cellular and serum TK1. There was no direct correlation between serum TK1 activity and protein level. It appears that a substantial fraction of serum TK1 is not enzymatically active.

**Conclusions:**

These results suggest that the serum TK1 protein differs from cellular or recombinant forms, is more active in high molecular weight complexes, and is sensitive to reducing agents. The results presented here provide important information for the future development and use of serum TK1 as a diagnostic biomarker in human and veterinary medicine.

## Background

Thymidine kinase (ATP: thymidine 5'- phosphotransferase, EC 2.7.1.21) is an enzyme involved in DNA precursor synthesis. It phosphorylates thymidine to dTMP, which is then further phosphorylated to thymidine diphosphate (dTDP) and triphosphate (dTTP) [1].

Thymidine kinase 1 (TK1) shows an S-phase dependent expression; TK1 activity increases in late G1 phase, reaches peak values during the S phase, and then decreases in the M phase. Accordingly, high levels of TK1 are found in proliferating and malignant cells and tissues [2-4]. Human TK1 has been extensively studied [5-7] and native human TK1 is found in two forms, a dimer form with low thymidine affinity (K_m_ = 15 μM) and a tetramer form with high thymidine affinity (K_m_ = 0.7 μM). ATP is a positive regulator in the tetramerization of human TK1 [6]. Recent structural studies of a TK1-like enzyme suggest that the binding of ATP leads to reorganization of the enzyme quaternary structure [8,9].

A form of TK1 is found at high levels in the sera of humans and animals with malignant tumors; therefore, serum TK1 activity has been used as a prognostic marker for several different tumor types, but primarily in leukemia and lymphoma [10-12].

Serum TK1 activity can be measured using a radioactive substrate analogue (Prolifigen TK-REA) [13] and this radio-enzymatic assay has been used in dogs with malignant lymphoma to predict the relapse of disease and to follow up therapy [14]. A non-radiometric TK1 activity assay (the TK Liaison assay, DiaSorin Inc.) is also used. Both assays provide clinically valuable information in humans and dogs with leukemia and lymphoma, particularly for monitoring therapy and predicting relapse [15]. The recent development of antibodies against human TK1 has enabled the determination of serum TK1 protein levels in different hematologic and solid tumors such as bladder carcinoma [16], breast carcinomas [17], and non-small cell lung cancer [18].

A basic question in this field concerns the structure of serum TK1 as compared to the cytosolic and recombinant enzyme. There is only one study that describes the molecular forms of human TK1 in serum [19]. It has been shown that serum TK1 activity occurs in many forms with molecular weights ranging from 60 kDa to 730 kDa. Treatment of serum with high concentrations of the reducing agent dithioerythritol (DTE) in the presence of 1 mM ATP reduced the molecular weight (MW) of the enzyme complexes from approximately 730 kDa to 100–200 kDa [19].

In the present study, full-length canine TK1 was cloned, expressed in *E. coli*, purified, and characterized. Human TK1 was also cloned and expressed in the same system in order to compare these two enzymes. The subunit structures of recombinant, cytosolic, and serum TK1 were determined by size exclusion chromatography and immunoaffinity methods.

## Methods

### Cloning and expression of full-length canine and human TK

The open reading frame of canine TK1 cDNA is 729 bp long, encoding 242 amino acids (Accession no XM-540462). Canine TK1 cDNA was PCR-amplified using an Incyte canine cDNA clone as the template (Open Biosystems, Lafayette, CO, USA). The amplified PCR fragment (729 bp) was cloned into the pEXP-5NT/TOPO vector (Invitrogen, Carlsbad, CA, USA) and sequenced. Full-length hTK1 cDNA was also cloned into the same vector and sequenced. The recombinant enzymes were purified by metal affinity chromatography on Ni-Sepharose resin as previously described [20].

### Cell culture and preparation of cell extracts

CCRF-CEM cells (human T lymphocyte cell line) were grown in Dulbecco’s Modified Eagle’s medium supplemented with 10 % newborn calf serum, 1 mM L-glutamine and PEST (2.4 IU penicillin and 2.4 μg/ml streptomycin). Madin Darby Canine Kidney (MDCK) cells (American Type Culture Collection, ATCC, Manassas, VA, USA) were cultured in ATCC-formulated Eagle’s Minimum Essential Medium supplemented with 10% newborn calf serum.

Cells (30–100 × 10^6^) were lysed in buffer containing 10 mM Tris/HCl, pH 7.6, 13.7 mM NaCl, 7 mM EDTA, 0.5% NP-40, 2 mM 4-2(aminoethyl)benzenesulfonyl fluoride hydrochloride (Pefabloc, Fluka) for 30 minutes at 4°C. The suspension was centrifuged at 16700 × g for 20 minutes at 4°C, and then the supernatant was stored in aliquots at −70°C after the addition of 20% glycerol.

### Anti- TK1 antibodies

**Figure 1:**
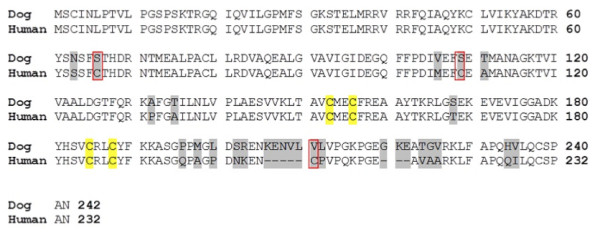
**Amino acid sequence alignment of canine and human TK1.** Residues that differ between the two sequences are shaded. Cysteines that are present in the human but not in the canine sequence are marked with squares. The Zn-binding cysteines are shaded in yellow. GenBank accession numbers are XM_540461 (dog) and KO_2582 (human).

Two rabbit polyclonal anti-dog TK1 antibodies were produced, one using a 28-amino acid (28-mer) synthetic peptide (amino acids 195–223) as the antigen by Agrisera AB (Umeå, Sweden), and the other one using a 16-amino acid (16-mer) synthetic peptide (211–225) as the antigen (Figure 1) by GenScript (Piscataway, NJ, USA).

The 31-mer mouse monoclonal anti-human TK1 antibody was produced against a 31-amino acid synthetic peptide (amino acids 194–225) (Figure 1) [3].

Sera from dogs with hematologic tumors were collected at the Small Animal Clinic, Department of Clinical Sciences, Swedish University of Agricultural Sciences, Uppsala, and approved by the Swedish Animal Ethics Committee. Human samples were collected at the Academic Hospital, Department of Laboratory Sciences, Uppsala, Sweden, and approved by the Local Ethics Committee.

### Thymidine kinase assay

TK1 activity was measured by a radiochemical method using the DE-81 filter paper technique as previously described [5]. [Methyl-^3^ H]-dThd (20 Ci/mmol, PerkinElmer) or ^3^ H]-3’-AZT (12 Ci/mmol, Moravek Biochemicals Inc, Brea, CA) was used as the labeled substrate. The standard reaction mixture contained 20 mM Tris–HCl pH 7.6, 2 mM dithiothreitol (DTT), 5 mM MgCl_2_, 5 mM ATP, 1% bovine serum albumin, and different concentrations of labeled substrate (ranging from 0.375 μM to 12.5 μM) in a final volume of 50 μl. One nanogram of recombinant TK1 was used in each reaction. The enzymes were diluted from a stock solution in Tris-buffered saline (pH 7.6) and 50% glycerol to 100 μg/ml before use. The specific activity was expressed as μmol/min/mg. One unit is defined as 1 μmol of dTMP/AZTMP formed per min and mg protein.

Serum TK1 activity was measured as previously described [21].

### Size exclusion chromatography

Size exclusion chromatography was performed essentially as described [19] using a Superose 12 column (1.0 × 30 cm, GE Healthcare, Sweden) attached to a fast protein liquid chromatography (FPLC) instrument (GE Healthcare). Crude cell extracts and sera (200 μl), diluted 1:1 in 0.01 M HEPES/KOH pH 7.6, 0.15 M NH_4_Cl, and 0.02% NaN_3_, were injected into the column equilibrated with the same buffer, and eluted at a flow rate of 0.4 ml/min. Fractions (0.4 ml) were collected and analyzed. The column was calibrated with standard proteins: α2-macroglobulin (720 kDa), β-amylase (200 kDa), bovine serum albumin (66 kDa), ovalbumin (45 kDa), and horse myosin (17 kDa).

### Immunoaffinity detection of TK1

Cell extracts and recombinant TK fractions were precipitated with 10% trichloroacetic acid (TCA) before western blot.

Polyclonal anti-dog TK1 antibody (16-mer) and the mouse monoclonal anti-human TK1 31-mer antibody were covalently coupled with CNBR-activated Sepharose 4B as described by the manufacturer (GE Healthcare). FPLC fractions (300 μl) from serum samples were diluted with 200 μl TBS and incubated with anti-dog TK1 or anti-human TK1 antibody-coupled Sepharose (50 μl). The samples were then agitated at 4°C for 4 hours followed by centrifugation. The antibody-coupled Sepharose was washed twice with TBS, once with TBS-T, and once again with TBS. Then, 40 μl sample buffer (containing 0.5 M Tris–HCl, pH 6.8, 20% glycerol, 10% (w/v) SDS, 0.1% bromophenol blue, and 10 mM DTT) was added to the Sepharose and incubated at room temperature for 20 minutes. The samples were then heated at 95°C for 5 minutes, and the proteins were resolved by 12% SDS-PAGE and transferred to a polyvinyl difluoride (PVDF) membrane (GE Healthcare) by a semi-dry transfer method as described by Invitrogen. The membranes were probed with a polyclonal anti-dog TK1 antibody (28-mer) or a chicken anti-human antibody (31-mer) and immune-reactive polypeptides were detected by the enhanced chemiluminescence method (GE Healthcare).

### Data analysis

The unpaired *t*-test was used to analyze the data, and a difference was regarded as significant when the p-value < 0.05. Kinetic parameters were obtained by fitting activity data using the Michaelis-Menten equation with the Sigma Plot Enzyme Kinetics program (SPSS Inc., Chicago, IL, USA).

## Results

### Cloning and expression of dog and human TK1

The canine and human TK1 amino acid sequences share 89.3% identity overall. The N-terminal 62 amino acids are identical, and the two sequences diverge mainly at the C-terminus, with only 59.5% identity in the last 41 amino acid residues (Figure 1); these residues are not present in the TK1 structures determined so far [7-9]. Another difference between these two sequences is that there are 11 cysteine residues in the human and 8 in the canine sequence; these may be involved in subunit interactions and may contribute to sensitivity to oxidizing and reducing agents.

Full-length canine and human TK1 cDNAs were cloned and expressed as fusion proteins with an N-terminal 6× histidine (His) tag and a TEV site, providing a 15-amino acid extension to the recombinant protein. Expression and purification of the His-tagged proteins were carried out as previously described [20]. The yields of dog and human TK1 were ~7 mg per liter of culture. The purity of the final recombinant TK1 proteins was >95% based on SDS-PAGE analysis.

### Substrate specificity, thermal stability, and pH dependence of recombinant dog and human TK1

**Figure 2:**
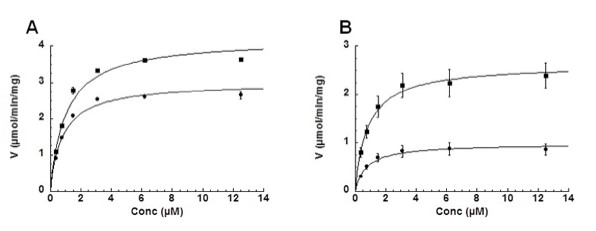
**Steady state kinetics of recombinant canine and human TK1.** Substrate saturation curve of canine **(A)** and human **(B)** TK1 with dThd (■) and AZT (●) as the variable substrate. Activity data were fitted to the Michaelis-Menten equation. The assays were repeated three times and error bars are shown.

The substrate specificity and stability of purified recombinant canine TK1 was characterized. Recombinant human TK1 was also characterized in parallel in order to compare the two enzymes. Steady-state kinetic analysis was performed in the presence of an excess ATP and variable concentrations of dThd or AZT, and the data were fitted to the Michaelis-Menten equation. The K_m_ values for dThd and AZT were 0.9 μM and 0.7 μM, respectively, for canine TK1 (Figure 2A), and 0.8 μM and 0.65 μM, respectively, for human TK1 (Figure 2B). Canine TK1 had a higher turnover rate (k_cat_) for both dThd and AZT than did human TK1. The efficiency of AZT phosphorylation (i.e., the k_cat_/K_m_ value) was 3-fold higher for canine TK1 than for human TK1 (Table 1). Both enzymes showed positive cooperativity with dThd and AZT, with Hill coefficients of 1.3 and 1.4, respectively (Table 1). The K_m_ values for ATP were 331 μM for dog TK1 and 234 μM for human TK1. Both enzymes also exhibited positive cooperativity with ATP, with Hill coefficients of 2.4 for canine TK1 and 2.3 for human TK1 (Table 1). When GTP, CTP, or UTP was used as the phosphate donor the activity was only about 3–10% of the activity seen with ATP for both enzymes (data not shown). Thymidine triphosphate (dTTP) acted as an efficient feedback inhibitor, with a relative IC_50_ value of 16 μM for canine TK1 and 23 μM for human TK1 when assayed with 1 μM dThd and 1 mM ATP (data not shown).

**Table 1:** Kinetic parameters of dog and human recombinant TK1

	*K*_*m*_*(μM)*	*V*_*max*_*(μmol/min/mg)*	*k*_*cat*_*(s*^*-1*^*)*	*k*_*cat*_*/K*_*m*_*(M*^*-1*^ *s*^*-1*^ *× 10*^*-6*^*)*	n*
*dTK1*					
*dThd*	0.88 ± 0.1	4.1 ± 0.1	1.81 ± 0.04	2.05	1.3 ± 0.1
*AZT*	0.70 ± 0.1	3.0 ± 0.1	1.32 ± 0.04	1.89	1.4 ± 0.2
*ATP*	331 ± 1.0	4.2 ± 0.3	1.85 ± 0.13	0.006	2.4 ± 0.4
*hTK1*					
*dThd*	0.83 ± 0.2	2.5 ± 0.2	1.06 ± 0.08	1.40	1.3 ± 0.4
*AZT*	0.70 ± 0.2	0.95 ± 0.06	0.40 ± 0.02	0.62	1.4 ± 0.2
*ATP*	243	2.3	0.98	0.004	2.3

Results are mean ± standard error of triplicate measurements. Data for human TK1 with ATP are from one typical experiment. * n Hill coefficient.

**Figure 3:**
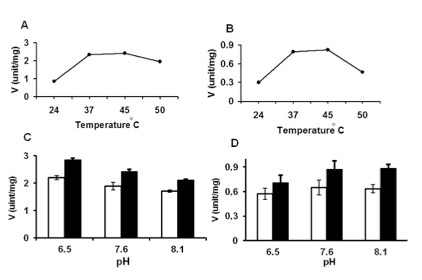
**Temperature and pH effects on the activities of recombinant canine and human TK1.** The activities of dog **(A)** and human **(B)** TK1 were measured using [^3^ H]-AZT as a substrate at different temperatures. The activities of canine **(C)** and human **(D)** TK1 were measured with AZT as substrate at different pH (indicated on the x-axis) and different temperatures: 37 °C (filled bars) and 45 °C (open bars).

The thermal stability of the two enzymes was determined at 24, 37, 45, and 50°C, and the optimum temperatures ranged from 37–45°C (Figure 3). The activity at 50°C was ~16% lower than that measured at 37°C for canine TK1, and ~40% lower for human TK1 (Figure 3). The effects of pH were evaluated using different buffers (i.e., Tris-Acetate pH 6.5, Tris–HCl pH 7.6, and HEPES/KOH buffer pH 8.1), and there were no significant differences in the activities of canine and human recombinant TK1 at these pH values at either 37 or 45°C (Figure 3).

The effect of a reducing agent (DTE) on the activities of both canine and human TK1 was also investigated; pre-incubation of the recombinant enzymes (40 ng/ml) with DTE at 0, 2, or 20 mM on ice for 30 min in the absence or presence of 1 mM ATP had no significant effect on the activities of both enzymes (p > 0.05). However, when the enzymes were incubated with 100 mM DTE (±ATP), the activity of both enzymes decreased significantly (60% for canine TK1, and 52% for human TK1; p < 0.05), suggesting that extremely high concentrations of the reducing agent (e.g., ≥100 mM DTE) may cause unfolding and loss of activity.

### Size distribution and the effect of reducing agent on recombinant TK1

**Figure 4:**
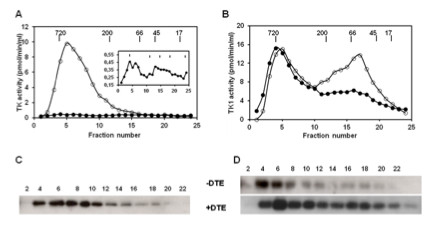
**Quaternary structure of recombinant TK1 and the effect of reducing agent.** Untreated (●) or DTE-treated (○) recombinant canine **(A)** and human **(B)** TK1 was analyzed by size exclusion chromatography. Four micrograms of freshly isolated TK1 in 200 μl buffer were injected into the column and eluted. A total of 24 fractions were collected. TK1 activity in the fractions was determined by radiochemical assay and TK1 protein was detected by immunoaffinity detection method as described in Materials and Methods. Inset: activity profile of untreated recombinant canine TK1. Arrows indicate the elution position of molecular weight markers. Western blot analysis of FPLC fractions of canine **(C)** and human **(D)** TK1; untreated (−DTE), and DTE-treated (+DTE). The numbers represent FPLC fractions.

To study the subunit composition of active TK1, recombinant canine and human TK1 were subjected to size-exclusion chromatography using a Superose 12 column in the absence of reducing agent in the buffer, in order to mimic the situation in blood, and to study the effects of reducing agent on subunit interactions. Four micrograms of freshly isolated recombinant TK1 were injected into the column and eluted. A total of 24 fractions were collected and assayed for TK1 activity, and the level of TK1 protein in each fraction was determined by western blot using a rabbit polyclonal antibody against canine TK1 or a mouse monoclonal antibody against human TK1 [3]. The recovery of recombinant canine TK1 was very low with activities ranging from 0.01–0.42 pmol/min/ml in the collected fractions (Figure 4A, insert), and no protein bands could be detected in any of the fractions by western blot analysis. However, when the same amount of recombinant canine TK1 was pre-incubated with 20 mM DTE for 30 min on ice, about 85% of the total activity was recovered in the fractions corresponding to the high MW range 300–720 kDa (Figure 4A). The canine TK1 protein in these fractions, detected by western blot analysis, correlated with the activity profile (Figure 4C). Currently, it is not clear why the recovery of the recombinant canine TK1 in the absence of DTE was so low, but one possible explanation is that a large fraction of the enzyme precipitated during chromatography. Nonetheless, reducing agents are apparently required for maintaining the activity of recombinant canine TK1.

For recombinant human TK1, about 60% of the activity was recovered in the MW range of 300–720 kDa, and about 40% in the range of 200–50 kDa (Figure 4B). Western blot analysis showed bands at 25 kDa in the fractions with TK1 activity (Figure 4D). Bands at 50 kDa were also detected in these fractions (data not shown). Pre-treatment with DTE led to a shift towards lower MW, with 56% of the TK1 activity eluting at 50–200 kDa range, whereas 47% eluted as high MW form of 300–720 kDa (Figure 4B). The protein levels, detected by western blot, correlated with the activity in these fractions (Figure 4D). Thus, the presence of DTE reduced the amount of the high MW forms of recombinant TK1, and increased the dimer and tetramer forms, suggesting that disulfide bonds are involved. These results demonstrate a significant difference between canine and human recombinant TK1 regarding their sensitivity to reducing agents.

### Cellular TK1 is active mainly as dimer and tetramer

**Figure 5:**
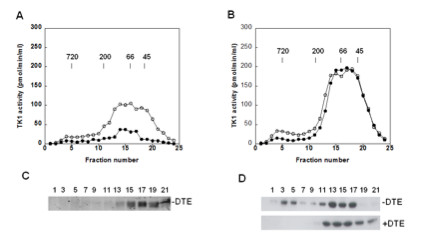
**Size distribution of cytosolic TK1.** TK1 activity in each fraction was measured using [^3^ H]-dThd as substrate. MDCK extracts **(A)** and CEM extracts **(B)** analyzed directly (○) or treated with DTE (●) prior to chromatography. Arrows indicate the elution position of the molecular weight markers. Western blot analyses of cytosolic canine TK1 **(C)** and human TK1 **(D)** in the FPLC fractions using polyclonal anti-dog TK1 antibody or mouse monoclonal anti-TK1 antibody for detection. Pre-treatment with DTE is indicated as + DTE. The numbers represent FPLC fractions.

Crude MDCK cell extracts were subjected to size-exclusion chromatography and the native canine TK1 eluted as a broad peak with MWs of 40–100 kDa. There was also some activity in the fractions at the higher MW range (Figure 5A). Western blot analysis showed a canine TK1 band (26 kDa) predominantly in fractions corresponding to MW of 40–100 kDa (Figure 5C).Treatment of the MDCK extracts with DTE prior to chromatography led to a significant decrease in TK1 activities in the corresponding fractions (Figure 5A). No TK1 bands were detected when extracts were pretreated with DTE (data not shown).

Crude CEM cell extracts were also analyzed by size-exclusion chromatography, and human TK1 activity was detected in fractions with MWs ranging from 40–720 kDa with a minor peak at 500–720 kDa and a major peak at 40–100 kDa (Figure 5B). Western blotting analysis showed a clear human TK1 band (25 kDa) in fractions corresponding to MWs of 40–100 kDa and 500–720 kDa (Figure 5D). When the same crude CEM extracts were incubated with DTE prior to chromatography, the level of TK1 activity eluted at high MW was reduced, and there was no change in the elution profiles (Figure 5B). Human TK1 (25 kDa) protein was detected only in fractions corresponding to the major peak (40–100 kDa) (Figure 5D). Thus, cellular (native) canine and human TK1 activity and protein are closely correlated, and both enzymes are present mainly as dimers and tetramers. Pretreatment with reducing agents inhibited canine TK1 activity but had little effect on human TK1 activity. These results are similar to previous reports [5,6].

### Oligomeric structures of serum TK1 and the effect of reducing agent

**Figure 6:**
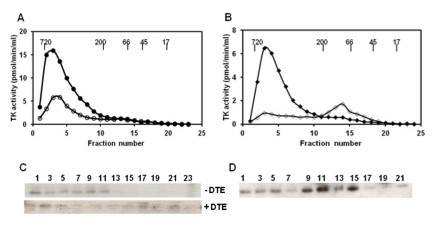
**Quaternary structures of serum TK1. (A)** Thymidine kinase activity in serum fractions from dogs with acute lymphocytic leukemia (●) injected directly into the Superose 12 column, or pre-treated with 20 mM DTE (○). **(B)** Thymidine kinase activity in serum fractions from human patients with acute lymphocytic leukemia (♦) injected directly into Superose 12 or pre-treated with DTE (◊). Arrows indicate the elution position of molecular weight markers. **(C)** Western blot analyses of untreated dog serum samples (−DTE) or those pre-treated with 20 mM DTE (+DTE), using polyclonal anti-dog TK1 antibody. **(D)** Western blot analyses of untreated human serum samples (−DTE) or those pre-treated with DTE (+DTE), using monoclonal anti-human TK1 antibody. The numbers represent FPLC fractions.

Sera from dogs with hematologic malignancies were applied to the Superose 12 column. TK1 activity and protein levels in the collected fractions were determined by a radiochemical assay and the immunoaffinity method. About 90% of total TK1 activity was recovered in fractions 1–9, corresponding to a MW range of 300–720 kDa. A polypeptide with an apparent MW of 26 kDa was detected in fractions 1–11 and the intensity of the bands did not correlate with activity levels in these fractions (Figure 6A and C). When the same sera were treated with DTE (20 mM) prior to chromatography, a reduction in TK1 activity was observed (Figure 6A), and the canine TK1 protein was detected in fractions corresponding to high MW but also in fractions corresponding to 25–66 kDa (Figure 6C).

A serum sample from dogs with lymphoma with low TK1 activity (3 pmole/min/ml) was also analyzed. Only one activity peak was observed at the high MW and no TK1 protein could be detected in the western blot analysis (data not shown).

Sera from human patients with acute lymphocytic leukemia were pooled and analyzed. Approximately 90% of the total TK1 activity eluted in fractions 1–9, corresponding to the MW range 300–720 kDa (Figure 6B). A polypeptide with an apparent MW of 25 kDa was detected in all fractions, and appeared as two peaks, one in fractions 1–5 and the other in fractions 9–15 (Figure 6D). When the same sample was pre-treated with DTE, TK1 activity was reduced in the corresponding fractions and the major activity peak shifted to the MW range 50–200 kDa (Figure 6B). No TK1 protein bands could be detected (data not shown).

A serum sample from human patients with relatively low TK1 activity (4 pmole/min/ml) was also analyzed. Only one activity peak was detected in the high MW fractions and no TK1 protein could be detected in any of the fractions (data not shown).

These results indicated that both dog and human serum TK1 are present in multimeric forms, as dimers, tetramers, and higher oligomers, and that the activities of both canine and human serum TK1 do not correlate with the level of TK1 protein in the serum. The serum enzyme is apparently more active in the multimeric complex form. Furthermore, treatment with high concentrations of DTE reduces the size of the complex but also leads to reduced activity, indicating that serum TK1 is different from recombinant TK1.

## Discussion

Recently serum TK1 activity has been used as biomarker for the diagnosis and prognosis of canine malignant lymphoma and leukemia [11,15,22,23]. However, the canine TK1 enzyme has not been characterized. Therefore, the full-length canine TK1 cDNA was cloned and expressed in *E. coli.* Recombinant canine TK1 was purified and characterized, and compared with human TK1, which was cloned and purified using the same procedures.

The kinetic properties of canine and human TK1 with their natural substrate Thd, ATP, and the anti-HIV nucleoside analog (e.g., AZT) were investigated. A reason for testing AZT is that it is used in the commercial TK1 Liaison assay. Canine TK1 had higher V_max_ values for all tested substrates than did human TK1. Therefore, the overall efficiency of canine TK1 was higher than that of human TK1. The high stability and efficiency of canine TK1 when using AZT as a substrate explains why the TK Liaison assay is suitable for canine lymphoma and leukemia studies [15,23].

The main focus of this study was to compare the quaternary structures of serum TK1 with those of cellular and recombinant enzymes, and to investigate the effect of reducing agents on the respective subunit compositions. Earlier studies have shown that native and recombinant human TK1 occur as tetramers in the presence of ATP or at high concentrations, and as dimers in the absence of ATP or at low enzyme concentrations [24,25]. Using similar techniques, we observed that recombinant human TK1 is mainly present in high MW complexes in addition to dimers and tetramers, and that pretreatment with DTE increased the extent of dimer and tetramer forms. Recombinant canine TK1, on the other hand, appeared to require reducing agents for proper folding, since in the absence of DTE only a minor fraction of the analyzed protein was recovered in high MW fractions, whereas in samples pre-treated with DTE, about 85% of the activity was recovered in the high MW form. Both canine and human serum TK1 eluted mainly as high MW complexes, and the dimer and tetramer forms had very low activity. Pretreatment with DTE resulted in >3-fold lower activity. In the case of human serum TK1, pretreatment with DTE also increased the proportion of active dimer and tetramer forms. We found that serum TK1 activity is associated with the TK1 oligomer, and there was no apparent correlation between serum TK1 activity and protein levels.

However, cytosolic TK1 from cultured canine and human cells was found mainly in dimer and tetramer forms, similar to previous reports [24,25]. These results indicate that the discrepancy regarding recombinant TK1 quaternary structures in our study compared with previous reports is not due to technical reasons but may be due to the conditions used.

Human TK1 contains 11 cysteines and canine TK1 contains 8 cysteines, four of which coordinate with Zn, which leaves 7 and 4 cysteines, respectively, as free thiol groups. The structures of TK1-like enzymes, from human, bacterial, and viral origins, are all in tetrameric forms and there are no intramolecular disulfide bonds observed, since the enzymes were crystalized in reducing conditions (10 mM DTT) [7-9]. In solution, in the absence of reducing agent, it is possible that surface cysteines form S-S bridges between the monomers, thereby forming oligomers. However, the oxidation of other residues like tyrosine or methionine, which lead to the formation of high molecular weight aggregates, is also possible. If oligomers were formed entirely through disulfide bonds, they should be completely reduced to dimer or tetramer in the presence of high concentrations of reducing agent. The fact that both serum and recombinant TK1 are persistently present as oligomeric forms irrespective of the presence of reducing agents suggested that other mechanisms are involved. In the case of human TK1, disulfide bonds are most likely involved, since pre-incubation with DTE increased the extent of dimer and tetramer formation. Furthermore, recombinant dog TK1 activity increased upon pre-incubation with DTE, but there was no reduction in the oligomeric form, suggesting that the reduction of oxidized residues, not necessarily cysteines, helped the enzyme to fold into the active form.

In the case of serum TK1, pre-incubation of the serum samples with DTE resulted in lower overall recovery of TK1 activity, suggesting that serum TK1 may be covalently associated through disulfide bonds with other stimulating or stabilizing factors. However, elucidation of the exact mechanism requires further study.

## Conclusions

Serum TK1 is present in sub-picomolar concentrations in the blood of healthy individuals and is greatly increased in patients with malignant diseases. Therefore, sensitive laboratory methods are needed to obtain the specificity and sensitivity required to measure this protein. The complexity of the TK1 protein in serum is most likely a major contributing factor to the difficulty in developing effective determination methods. We found here that serum TK1 exists in a mixture of different molecular sizes, that only a fraction of the TK1 protein in serum is associated with TK1 activity, and that reducing agents have negative effects on serum TK1 activity. These results may help in the development of TK1 as a biomarker, both in human and veterinary medicine.

## Competing interests

S. Eriksson is the holder of a TK1 patent licensed to DiaSorin and is a shareholder in AroCell AB. None of the other authors has any financial or personal relationships with other people or organizations that could inappropriately influence or bias the paper.

## Authors’ contributions

HS performed the kinetics experiments, TK activity measurements in all samples, immunoblot analyses of the cellular enzyme, and statistical data analysis, and prepared the manuscript. KJ purified the polyclonal anti-dog TK1 antibodies and performed the immunoblot analyses of recombinant and serum enzyme samples. LW cloned, expressed, and purified the recombinant enzymes and critically revised the manuscript. EH contributed to the initial experimental design. SE designed the study and revised the manuscript. All authors have read and approved the final manuscript.
